# *MKRN2* inhibits migration and invasion of non-small-cell lung cancer by negatively regulating the PI3K/Akt pathway

**DOI:** 10.1186/s13046-018-0855-7

**Published:** 2018-08-13

**Authors:** Jun Jiang, Yitong Xu, Hongjiu Ren, Muli Wudu, Qiongzi Wang, Xin Song, Hongbo Su, Xizi Jiang, Lihong Jiang, Xueshan Qiu

**Affiliations:** 1 Department of Pathology, First Affiliated Hospital College and of Basic Medical Sciences China Medical University, No. 155 Nanjing North Street, Heping District, Shenyang, 110001 Liaoning China; 2 Jilin Zhongzheng Judicial Appraisal Institute, Changchun, China; 3 Department of Pathology, General Hospital of Liaohe Oil Field, Panjin, China

**Keywords:** Non-small-cell lung cancer, *MKRN2*, Cell migration, Cell invasion, PI3K/Akt pathway, Ubiquitination

## Abstract

**Background:**

Makorin RING zinc finger-2 (*MKRN2*) belongs to the makorin RING zinc finger family and is a novel ubiquitin E3 ligase targeting the p65 subunit of NF-κB to negatively regulate inflammatory responses; however, the relationship between *MKRN2* and tumorigenesis remains unclear. In this study, we clarified the role of *MKRN2* in non-small cell lung cancer (NSCLC).

**Methods:**

Tumor specimens collected from 261 NSCLC patients from 2013 to 2017 were retrieved from the Pathology Archive of the First Affiliated Hospital of China Medical University, and we performed assays to evaluate *MKRN2* expression and to determine the impact of *MKRN2* silencing and overexpression on NSCLC-cell migration and invasion.

**Results:**

We demonstrated that *MKRN2* expression was associated with lymph node metastasis, p-TNM stage, cancer-cell differentiation, and poor prognosis. By altering the expression of *MKRN2* in selected cell lines, we found that *MKRN2* inhibited cell migration and invasion through downregulation of the PI3K/Akt pathway.

**Conclusions:**

These results suggested that *MKRN2* inhibited NSCLC progression by reducing the metastatic potential of cancer cells. Our findings provide critical insight into the association of *MKRN2* expression with favorable clinicopathological characteristics in NSCLC patients and suggested that *MKRN2* plays a role in inhibiting NSCLC development.

## Background

Lung cancer is among the leading causes of all cancer-related deaths worldwide, and the incidence continues to increase. Approximately 85 to 90% of lung cancer cases are diagnosed as non-small-cell lung cancer (NSCLC) [1–3]. Overall, the 5-year survival rate has remained at 15%, with the prognosis of patients with NSCLC principally correlating with tumor metastasis associated with transcriptional regulation of certain critical genes [4–6]. Therefore, it is necessary to understand the mechanisms involved in NSCLC progression.

The phosphatidylinositol 3-kinase (PI3K)/Akt pathway is recognized as a key pathway in carcinogenesis and commonly identified in diverse human tumors [7]. The central role of PI3K/Akt signaling in this complex network of cellular processes makes this pathway highly important in cancer cells. Accordingly, phosphorylated (p-)Akt is overexpressed in a multitude of human cancers and related to poor overall survival in some cancer types [8–12]. Akt activation can be determined through the use of phospho-specific antibodies against either p-Akt (Ser473) or p-Akt (Thr308) [13]. Patterns of p-Akt staining vary between follicular and papillary carcinomas, but more prominent nuclear p-Akt (Ser473) has been reported in regions of invasion by both types [14]. Similarly, although differentiated thyroid cancers demonstrate cytoplasmic staining, contiguous anaplastic thyroid cancers display both nuclear and cytoplasmic p-Akt (Ser473) [15]. These findings might suggest a role for nuclear p-Akt in aggressive or invasive disease. Additionally, significant evidence suggests that the PI3K/Akt pathway plays critical roles in multiple tumors of endocrine tissues [16].

Makorin RING zinc finger-2 (*MKRN2*) belongs to the makorin RING zinc finger family, and the *MKRN2* gene partially overlaps the *RAF1* proto-oncogene in antisense transcriptional orientation [17]. This family encodes putative ribonucleoproteins with a distinctive array of zinc finger domains [18]. To date, nine MKRN-family loci at various sites in the human genome have been identified. Makorins are zinc finger proteins with a typical C3HC4 motif (the RING domain) associated with arrays of one to four C3H domains and representing a type of zinc finger found in a variety of ribonucleoproteins [19, 20]. *MKRN2* harbors four C3H zinc fingers and a signature C3HC4 RING zinc finger domain [21]. MKRN proteins also contain a protein-protein interaction motif rich in Cys and His residues, but that exhibits a currently unknown function specific to MKRNs [17]. This motif is found in most E3 ubiquitin ligases, a category of enzymes mediating the transfer of ubiquitin from an E2 ubiquitin-conjugating enzyme to target protein substrates. The RING domain is responsible for ubiquitin ligase activity, leading to monoubiquitination and/or to synthesis of polyubiquitin chains on lysine residues [22–24]. Accordingly, some MKRN proteins work as E3 ubiquitin ligases [25], with a previous study reporting that *MKRN2* is a novel ubiquitin E3 ligase targeting the p65 subunit of NF-κB to negatively regulate inflammatory responses [26].

In this study, we demonstrated that *MKRN2* inhibited cell migration and invasion of NSCLC cells by reducing the p-Akt (Ser473) levels. Additionally, we showed that *MKRN2* was involved in ubiquitin-dependent degradation of the p85α subunit of PI3K (PI3Kp85α). Moreover, we examined *MKRN2* expression in NSCLC tissues and cell lines by immunohistochemistry and western blot and altered *MKRN2* expression in these cells to evaluate changes in cancer-related phenotypes in order to determine its role in NSCLC.

## Methods

### Specimens and patient data

Tumor specimens collected from 261 patients with NSCLC from 2013 to 2017 were retrieved from the Pathology Archive of the First Affiliated Hospital of China Medical University (Shenyang, China). All enrolled patients underwent curative surgical resection without having prior chemotherapy or radiation therapy. This study was approved by the Medical Research Ethics Committee of China Medical University, and informed consent was obtained from all patients.

### Immunohistochemistry

All specimens were fixed in 10% neutral formalin, embedded in paraffin, and prepared as 4-μm-thick serial sections. Immunostaining was performed according to the streptavidin-peroxidase method. The sections were incubated with anti-*MKRN2* rabbit polyclonal antibodies (1:100; HPA037559; Sigma-Aldrich, Shanghai, China) at 4 °C overnight. Sections were washed in phosphate-buffered saline (PBS) and incubated with reagents A and B (EliVsion Reagent; KIT9921; MaiXin, Fuzhou, China), according to manufacturer instructions. Sections were then developed using 3,3-diaminobenzidine tetrahydrochloride (MaiXin), lightly counterstained with hematoxylin, dehydrated in alcohol, and mounted. Two investigators blinded to the clinical data semiquantitatively scored all slides by evaluating the staining intensity and percentages of cells stained in representative areas of each slide. The percentages of stained cells were scored as follows: 1 (1–25%), 2 (26–50%), 3 (51–75%), or 4 (76–100%). Based on the staining intensity, *MKRN2* expression was also scored as follows: 0 (no staining), 1 (weak staining), 2 (moderate staining), and 3 (high staining). Percentage scores were assigned as follows: 0 (0%), 1 (1–30%), 2 (31–70%), and 3 (71–100%). The scores of each tumor sample were multiplied to give a final score ranging from 0 to 9, with tumor samples scored > 3 considered *MKRN2*-high, and those scored ≤3 considered *MKRN2*-low [14].

### Cell culture

All cell lines were purchased from the Cell Bank of the China Academy of Sciences (Shanghai, China) and cultured in medium containing 10% qualified fetal calf serum (FB15015; Clark Biosciences, Richmond, VA, USA), 100 IU/mL penicillin (Sigma-Aldrich, St. Louis, MO, USA), and 100 μg/mL streptomycin (Sigma-Aldrich). HBE cells were cultured in Dulbecco’s modified Eagle medium (DMEM) with high glucose; A549, H1299, H460, H226, and H292 cells were cultured in Roswell Park Memorial Institute 1640 medium; and SK-MES-1 cells were cultured in MEM medium. Cells were maintained in a 5% CO_2_ incubator at 37°C [27].

### Plasmid construction and transfection and administration of PI3K/Akt inhibitors

Cell transfection was performed using Lipofectamine 3000 reagent (Invitrogen, Carlsbad, CA, USA) according to manufacturer instructions. In *MKRN2-*knockdown experiments, cells were transfected with *MKRN2*-specific small-interfering (si)RNA and scrambled control siRNA (Ribobio, Guangzhou, China) for 48 h. For *MKRN2* overexpression, cells were transfected with an *MKRN2*-expression plasmid and the corresponding empty EX05 vector (LongqianBiotech, Shanghai, China).

To inhibit PI3K/Akt signaling, cells were treated with 10 μM LY294002 (MedChemExpress, Monmouth Junction, NJ, USA), an inhibitor that blocks the PI3K/Akt pathway. LY294002 was dissolved in dimethyl sulfoxide (DMSO) and added 12 h after a 36-h transfection, with the same volume of DMSO added to control cells.

### Immunocytochemistry

Lung cancer cells cultured in 24-well plates for 24 h were fixed in paraformaldehyde (4%) for 15 min and permeabilized with 0.1% Triton X-100 for 10 min. After washing with PBS, cells were blocked in bovine serum albumin (5%) for 1 h and incubated with the anti-*MKRN2* antibody (1:100) for 16 h at 4 °C, followed by incubation with a tetramethylrhodamine-conjugated secondary antibody for 2 h. Nuclei were counterstained with 4′,6-diamidino-2-phenylindole for 10 min. Cell images were captured using an Olympus FV1000 laser-scanning confocal microscope (Olympus, Tokyo, Japan).

### Quantitative real-time polymerase chain reaction (PCR)

Quantitative real-time PCR was performed in a 7900HT fast real-time PCR system (Applied Biosystems, Foster City, CA, USA) using SYBR Green PCR master mix in a total volume of 20 μL under the following cycling conditions: 95°C for 30 s and 45 cycles of 95°C for 5 s and 60°C for 30 s. The dissociation step was used to generate a melting curve and confirm amplification specificity. Relative gene expression was calculated using the 2^−ΔΔCt^ method, with β-actin used as a reference. Primer sequences were as follows: *MKRN2* forward, 5′-AAGGCCTCTGCTTCTGAGAG-3′, and reverse, 5′-GATATCACACGGCATTCTGG-3′; and β-actin forward, 5′-ATAGCACAGCCTGGATAGCAACGTAC-3′, and reverse, 5′-CACCTTCTACAATGAGCTGCGTGTG -3′. All experiments were performed in triplicate.

### Western blot

Total cellular protein was extracted using lysis buffer (P0013; Beyotime Biosciences, Shanghai, China) containing a protease-inhibitor cocktail (B14002; Biotool, Shanghai, China) and in the presence or absence of a phosphatase-inhibitor cocktail (B15002; Biotool) according to manufacturer instructions and quantified using the Bradford method. Proteins (80 μg/lane) were separated by 10% sodium dodecyl sulfate polyacrylamide gel electrophoresis, transferred to polyvinylidene fluoride membranes (Millipore, Billerica, MA, USA), and the membranes were blocked in 5% skim milk (232,100; Becton Dickenson, Franklin Lakes, NJ, USA) in Tris-buffered saline with Tween-20 at room temperature, followed by incubation overnight at 4°C with the appropriate primary antibodies (Table 1). Membranes were washed and then incubated with horseradish peroxidase-conjugated anti-mouse/rabbit IgG (1:2000; ZSGB-BIO, Beijing, China) at 37°C for 2 h. Immune reactivity was detected by enhanced chemiluminescence (Thermo Fisher Scientific, Waltham, MA, USA) using a BioImaging system (UVP, Inc., Upland, CA, USA). Relative protein expression was calculated after normalization to glyceraldehyde 3-phosphate dehydrogenase (GAPDH), which was used as a loading control.

**Table 1:** List of antibodies used for western blot

Antibody name	Source	Catalog number	Host	Dilution
MKRN2	Sigma-Aldrich	HPA037559	Rabbit	1:100
GAPDH	Beyotime	AF0006	Mouse	1:1000
RhoA	Cell Signaling Technology	2117	Rabbit	1:500
MMP9	Cell Signaling Technology	13,667	Rabbit	1:500
ROCK1	Wanlei Bio.	Wl01761	Rabbit	1:500
PI3Kp85α	Santa Cruz Biotechnology	SC-423	Rabbit	1:100
PI3Kp85α	Cell Signaling Technology	4257	Rabbit	1:500
PI3Kp85α	Cell Signaling Technology	13,666	Mouse	1:50
AKT	Cell Signaling Technology	4685	Rabbit	1:1000
p-AKT(ser473)	Cell Signaling Technology	4060	Rabbit	1:500
IgG	Byotime	A7028	Mouse	1:50
HA	TransGen Biotech	HT301	Mouse	1:1000

### Co-immunoprecipitation assays

Assays were performed as described previously [28].

### Cell migration and invasion analyses

Cell migration and invasion assays were performed in 24-well Transwell chambers containing inserts with a pore size of 8 μm (Costar, Washington, DC, USA). For the invasion assay, inserts were coated with 100 μL Matrigel (1:9 dilution; BD Biosciences, San Jose, CA, USA). Cells were trypsinized 24 h after transfection, and 5 × 10^4^ cells in 100 μL medium supplemented with 2% fetal bovine serum (FBS) were transferred to the upper Transwell chamber, whereas 600 μL medium supplemented with 10% FBS was added to the lower chamber. After incubation for 24 h, cells on the upper membrane surface were removed with a cotton tip, and those passed through the membrane were fixed with paraformaldehyde and stained with hematoxylin. The number of migrated/invaded cells was counted in 10 randomly selected fields under a microscope at high magnification. All experiments were repeated independently at least three times under identical conditions.

### Proteasome-inhibition and ubiquitination assays and immunoprecipitation (IP)

For proteasome-inhibition assays, cells were transfected with the indicated plasmids, and at 48-h post-transfection, the 26S proteasome inhibitor MG132 (s1748; Beyotime Biosciences) was added at a final concentration of 10 μM for 5 h, after which samples were collected. Cells were lysed in lysis buffer, and cell debris was pelleted by centrifugation at 12,000 rpm for 10 min at 4 °C. Supernatants were collected for IP, which was performed using whole-cell lysates (~ 200 μg protein), 4 μg to 10 μg antibody, and 20 μL protein A/G agarose (P2012; Beyotime Biosciences). Cell lysates were precleared with 20 μL agarose A/G beads by rocking for 1 h at 4 °C, after which beads were removed, and appropriate antibodies were added. Samples were then incubated with 20 μL agarose A/G beads by rocking for 4 h to 6 h at 4 °C, followed by the addition of lysates and incubation with rocking overnight at 4 °C. Immune complexes were collected by centrifugation, followed by washing in cell lysis buffer before analysis. Levels of PI3Kp85α ubiquitination were evaluated by IP of PI3Kp85α using anti-PI3Kp85α antibodies, followed by anti-hemagglutinin (HA) immunoblotting.

### Statistical analysis

All statistical analyses were performed using SPSS 17.0 (SPSS, Inc., Chicago, IL, USA). Kaplan–Meier survival analyses were performed and compared using the log-rank test [29, 30]. Differences between groups were determined by Student’s *t* test, and a *P* < 0.05 was considered statistically significant.

## Results

### *MKRN2* is expressed at low levels in NSCLC cells and correlates with poor prognosis

**Fig. 1:**
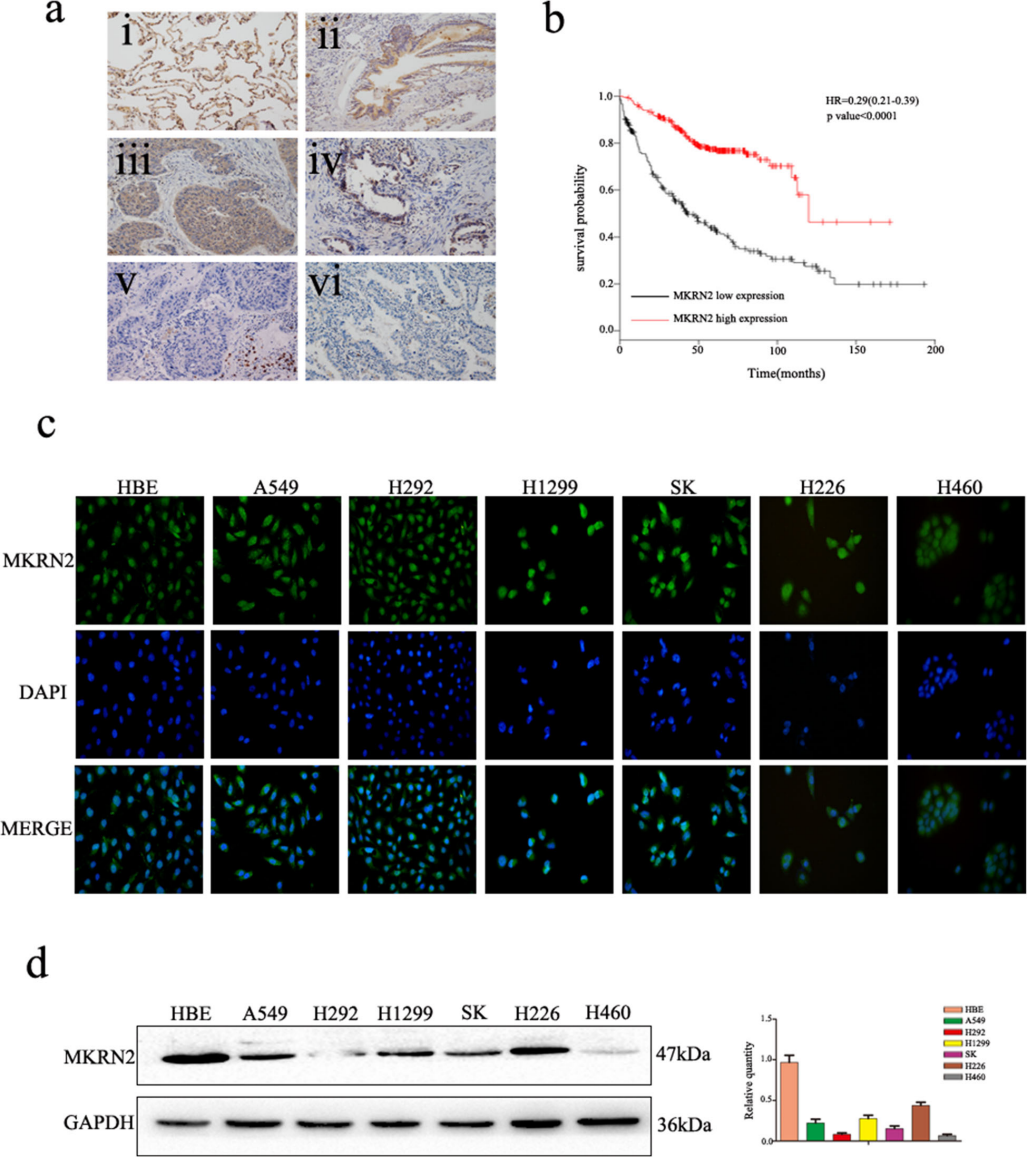
Expression of *MKRN2* in NSCLC is associated with poor clinical prognosis. **a** *MKRN2* protein level analyzed by immunohistochemistry in (i) alveolar and (ii) normal bronchial epithelial cells, (iii) well-differentiated squamous cell carcinoma and (iv) adenocarcinoma, and (v) poorly differentiated squamous cell carcinoma and (vi) adenocarcinoma. **b** Survival of NSCLC patients with high and low *MKRN2* expression analyzed based on Kaplan–Meier curves. Hazard ratio (HR) and *P* value are indicated. **c** Immunofluorescence assays were performed to detect *MKRN2* localization in human normal bronchial epithelial and NSCLC cell lines. *MKRN2* was located in the cytoplasm and nucleus. **d** MKRN2 levels in HBE cells and six NSCLC cell lines according to western blot analysis. Relative quantification analysis was based on grayscale values

Immunohistochemistry analyses of the 261 NSCLC tissues and immunofluorescence analyses of seven NSCLC cells revealed that *MKRN2* was localized in the cytoplasm and nucleus (Fig. 1a and c). Additionally, *MKRN2-*expression levels were significantly lower in NSCLC tissues relative to those in normal lung tissues, alveoli, and bronchial tissues. We then investigated the relationship between *MKRN2* protein level and clinical parameters. As shown in Table 2, *MKRN2* protein levels were negatively correlated with clinicopathological parameters, including differentiation (*P* < 0.001), lymph node metastasis (*P* = 0.002), and pathological TNM stage (*P* = 0.005), whereas correlations between *MKRN2* protein levels and tumor size (*P* = 0.936), histological type (*P* = 0.562), and patient age (*P* = 0.374) and sex (*P* = 0.318) were not statistically significant. Moreover, Kaplan–Meier analysis showed that high *MKRN2* levels were associated with higher rates of NSCLC-patient survival (Fig. 1b). These findings suggest that *MKRN2* might have an effect on lung cancer cell migration and invasion.

**Table 2:**
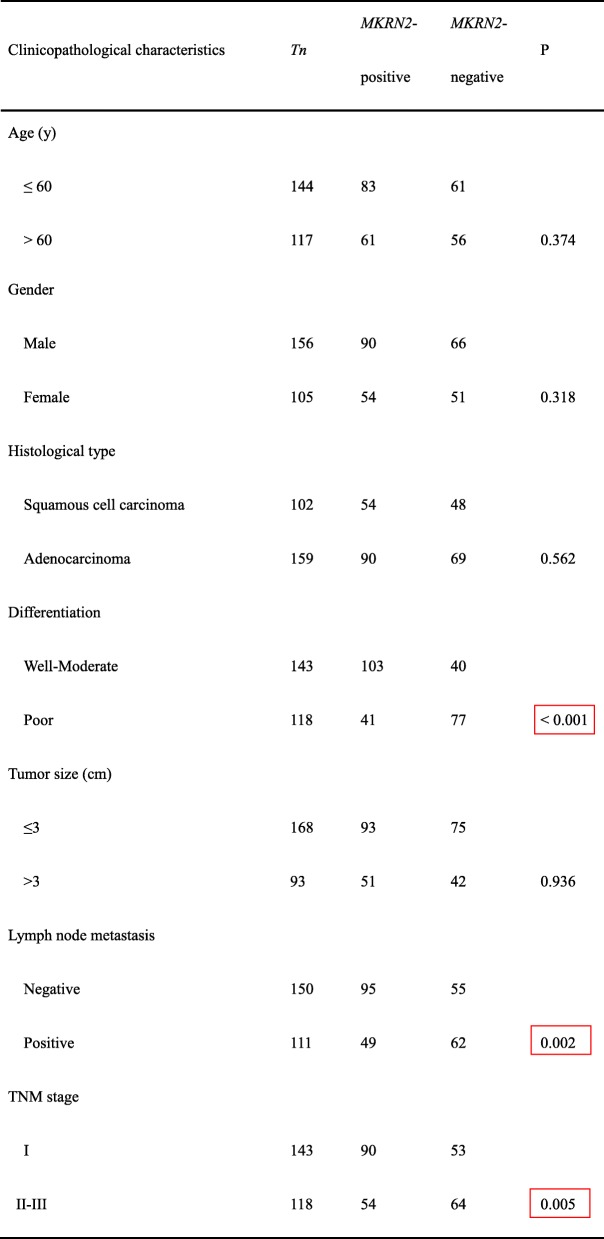
Association of *MKRN2* expression with clinical and pathological characteristics of NSCLC patients

We then performed western blot analysis to detect *MKRN2* protein levels in normal bronchial epithelial cells and commonly used NSCLC cell lines, finding that *MKRN2* protein levels were lower in tumor cell lines than in normal bronchial epithelial cells (Fig. 1d). Therefore, we choose A549 and H1299 cell lines, which showed moderate levels of *MKRN* expression, for subsequent experiments.

### *MKRN2* inhibits migration and invasion of NSCLC cells

**Fig. 2:**
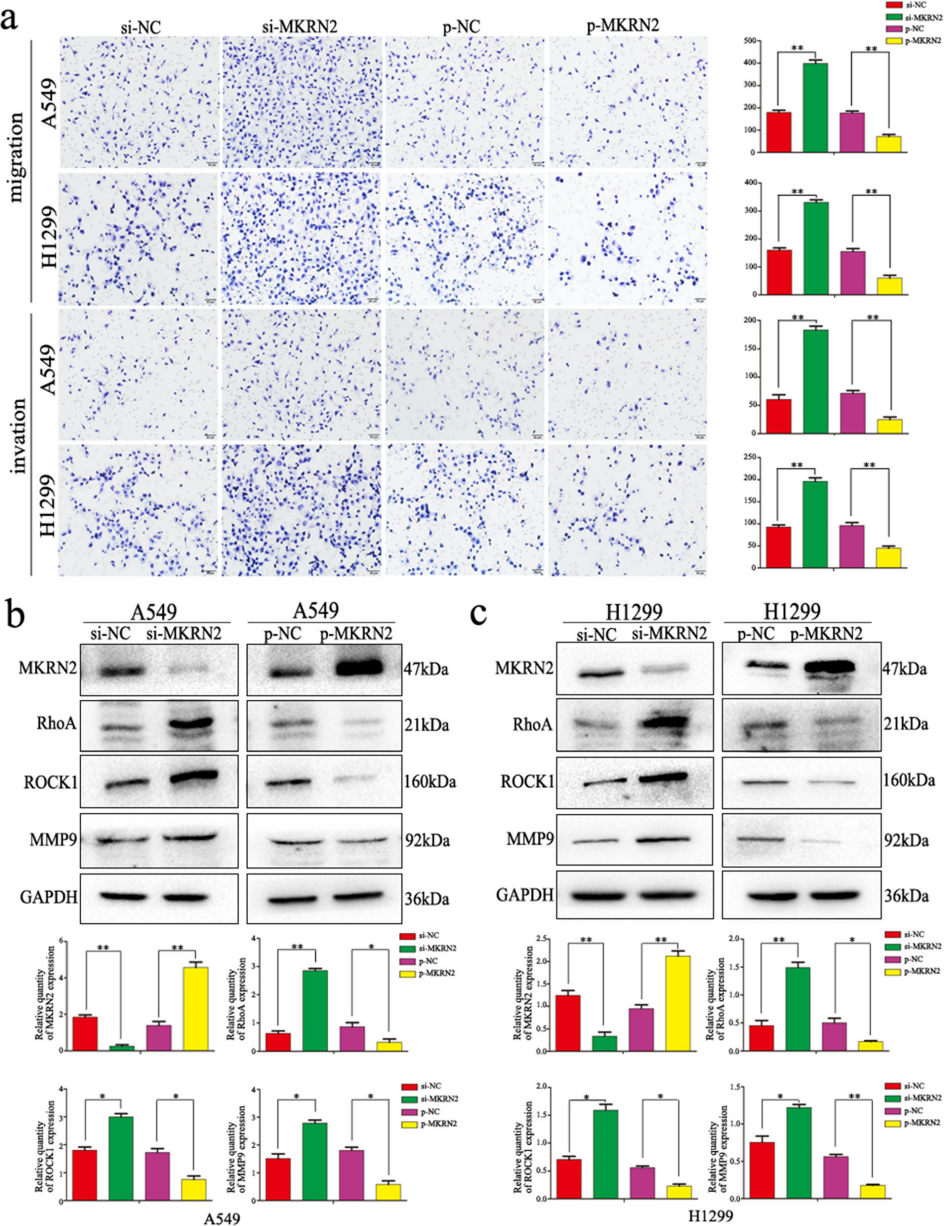
*MKRN2* protein levels are negatively correlated with the migration and invasion of NSCLC cells. **a** Transwell assays were performed to assess cell migration and invasion in the context of *MKRN2* overexpression and downregulation. *MKRN2* overexpression in A549 and H1299 cells inhibited cell migration and invasion, and *MKRN2* knockdown enhanced cell migration and invasion. *P < 0.05; **P < 0.01. **b, c** The effects of *MKRN2* levels on the expression of proteins associated with cell migration and invasion in transfected **b** A549 and **c** H1299 cells. Relative quantification analysis was based on grayscale values. *P < 0.05; **P < 0.01

Because *MKRN2* levels correlated with NSCLC progression, we explored the biological function of *MKRN2* in lung cancer cells by genetically manipulating *MKRN2* expression. We downregulated or upregulated *MKRN2* expression in A549 and H1299 cell lines using siRNA or the EX05-flag *MKRN2* vector, respectively. Transwell assays showed knockdown of *MKRN2* promoted the migration and invasion of A549 and H1299 cells as compared with control cells, whereas *MKRN2* overexpression inhibited this activity by both cell lines (*P* < 0.05) (Fig. 2a). Consistent with these results, variations in *MKRN2* protein levels affected the expression of proteins involved in cell migration and invasion, with levels of RhoA, Rho-associated protein kinase (ROCK)1, and matrix metalloproteinase (MMP)9 upregulated following *MKRN2* knockdown, whereas *MKRN2* overexpression caused the opposite effect (Fig. 2b and c). These findings demonstrated that *MKRN2* suppressed the migration and invasion of lung cancer cells, consistent with our immunohistochemical results.

### *MKRN2* regulates the migration and invasion of NSCLC cells via the PI3K/Akt pathway

**Fig. 3:**
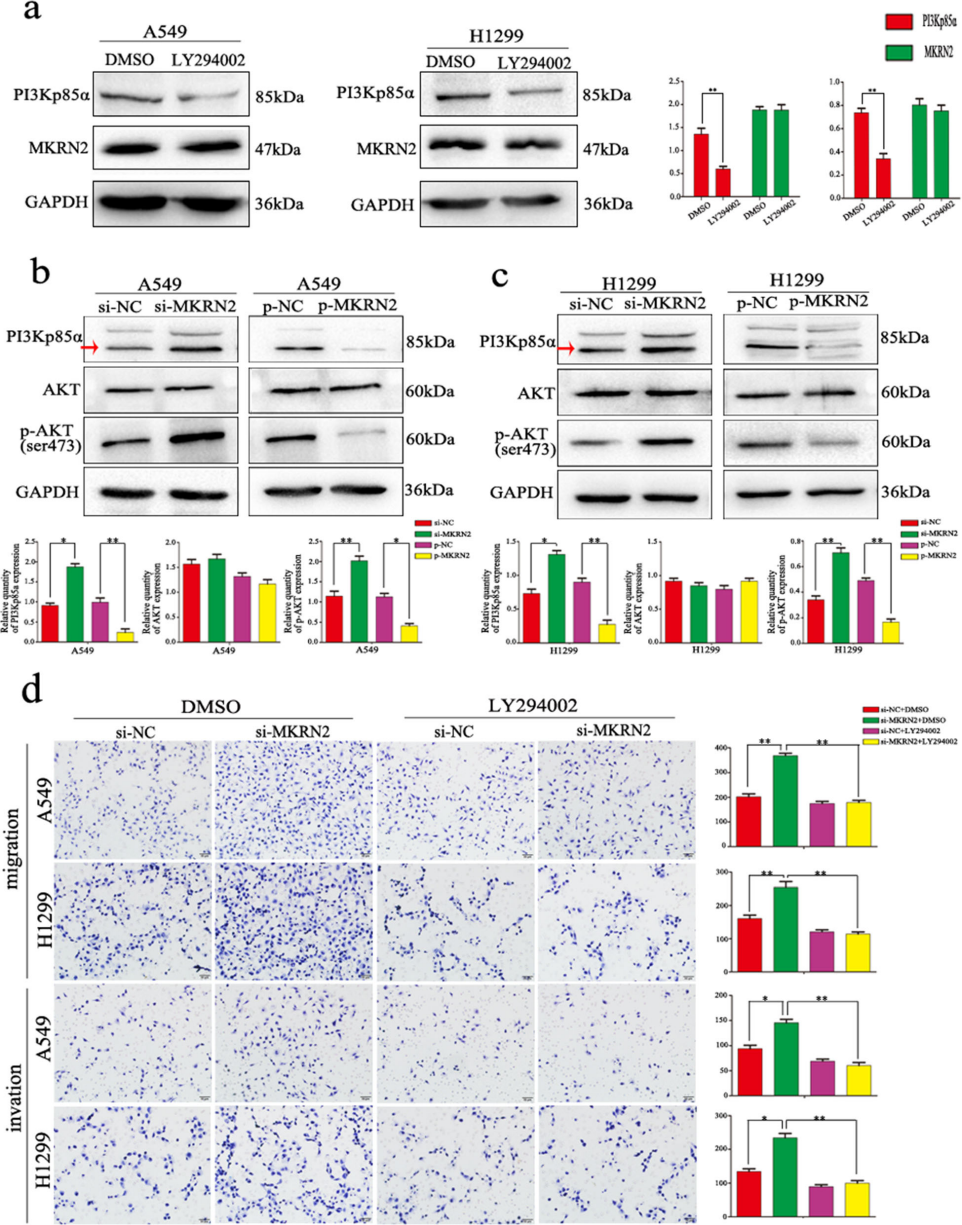
*MKRN2* regulates cell migration and invasion through the PI3K/Akt pathway. **a** *MKRN2* expression following knockdown of PI3K levels. **b, c** Changes in the expression of PI3K/Akt-related proteins in **b** A549 and **c** H1299 cells. Relative quantification analysis was based on grayscale values. *P < 0.05; **P < 0.01. **d** Changes in the migration and invasion activity of A549 and H1299 cells in the presence or absence of treatment with the PI3K inhibitor LY294002 and transfection with si-*MKRN2*. *P < 0.05; **P < 0.01

**Fig. 4:**
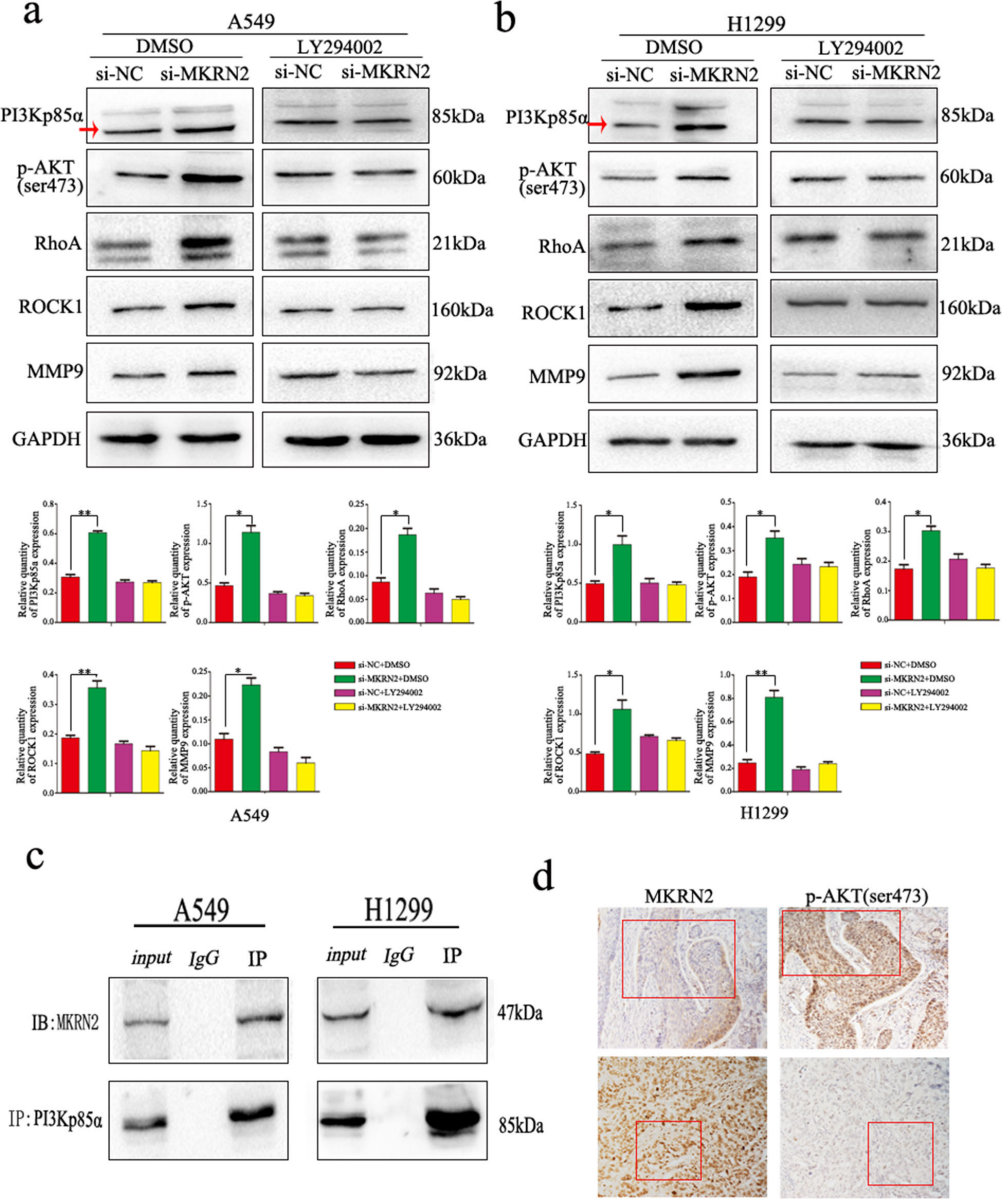
Effect of *MKRN2* in the presence or absence of PI3K inhibition on protein expression in NSCLC cells. **a** A549 and **b** H1299 cells transfected with si-*MKRN2* were treated or not treated with the PI3K inhibitor LY294002 analyzed by western blot for the expression of proteins involved in cell migration and invasion. Relative quantification analysis was based on grayscale values. *P < 0.05; **P < 0.01. **c** Interactions between *MKRN2* and PI3Kp85α in A549 and H1299 cells as measured by co-immunoprecipitation**. d** The relationship between *MKRN2* expression and p-Akt (Ser473) levels as analyzed by immunohistochemistry

Based on the relationships between lymph node metastasis and *MKRN2* expression in clinical cases and the functions of *MKRN2* in NSCLC cell migration and invasion, we investigated the specific biological mechanisms associated with these effects. Previous studies reported associations between *MKRN2* expression and the Akt-signaling pathway [31], specifically that *MKRN2* acts downstream of PI3K; therefore, we examined whether PI3Kp85α affects *MKRN2* expression, with results showing no changes in *MKRN2* expression following PI3Kp85α downregulation in A549 and H1299 cells (Fig. 3a). However, evaluation of the effects of *MKRN2* expression on activation of the Akt pathway in A549 and H1299 cells revealed that *MKRN2* knockdown and overexpression upregulated and downregulated levels of PI3Kp85α, respectively, and that *MKRN2* overexpression reduced Akt phosphorylation, whereas *MKRN2* knockdown increased Akt phosphorylation in A549 cells (Fig. 3b), with the same effects detected in H1299 cells (Fig. 3c). A possible explanation for the differences in results between the two studies could be that the role of the nervous system differs from that of the tumor. To investigate whether *MKRN2* function was mediated through the PI3K/Akt pathway, we evaluated the effects of this pathway using the inhibitor LY294002, which inhibits PI3K expression and reduces Akt phosphorylation. In A549 and H1299 cells transfected with si-*MKRN2*, LY294002 administration reduced the induction of cell migration and invasion (Fig. 3d), and the negative correlation between *MKRN2* expression and RhoA, ROCK1, and MMP9 levels was reversed (Fig. 4a and b). We then verified interactions between *MKRN2* and PI3Kp85α in A549 and H1299 cells by co-immunoprecipitation analysis (Fig. 4c), with results confirming this interaction. As shown in Table 3,immunohistochemistry of 34 pairs NSCLC tissues revealed that levels of p-Akt (Ser473) were low in cells exhibiting high levels of *MKRN2* expression, whereas p-Akt (Ser473) levels were high in cells exhibiting low levels of *MKRN2* expression (Fig. 4d), indicating a negatively correlated relationship between *MKRN2* expression and p-Akt (*P* = 0.03). These results suggested that activation of the PI3K/Akt pathway contributed to the *MKRN2*-mediated migration and invasion of NSCLC cells.

**Table 3:** Relationship between *MKRN2* expression and p-Akt levels in NSCLC patients

p-Akt	(+)	(−)	r	P
*MKRN2*				
(+)	8	9	−0.369	0.032
(−)	14	3		

### *MKRN2* is involved in the ubiquitin-dependent degradation of PI3Kp85α

**Fig. 5:**
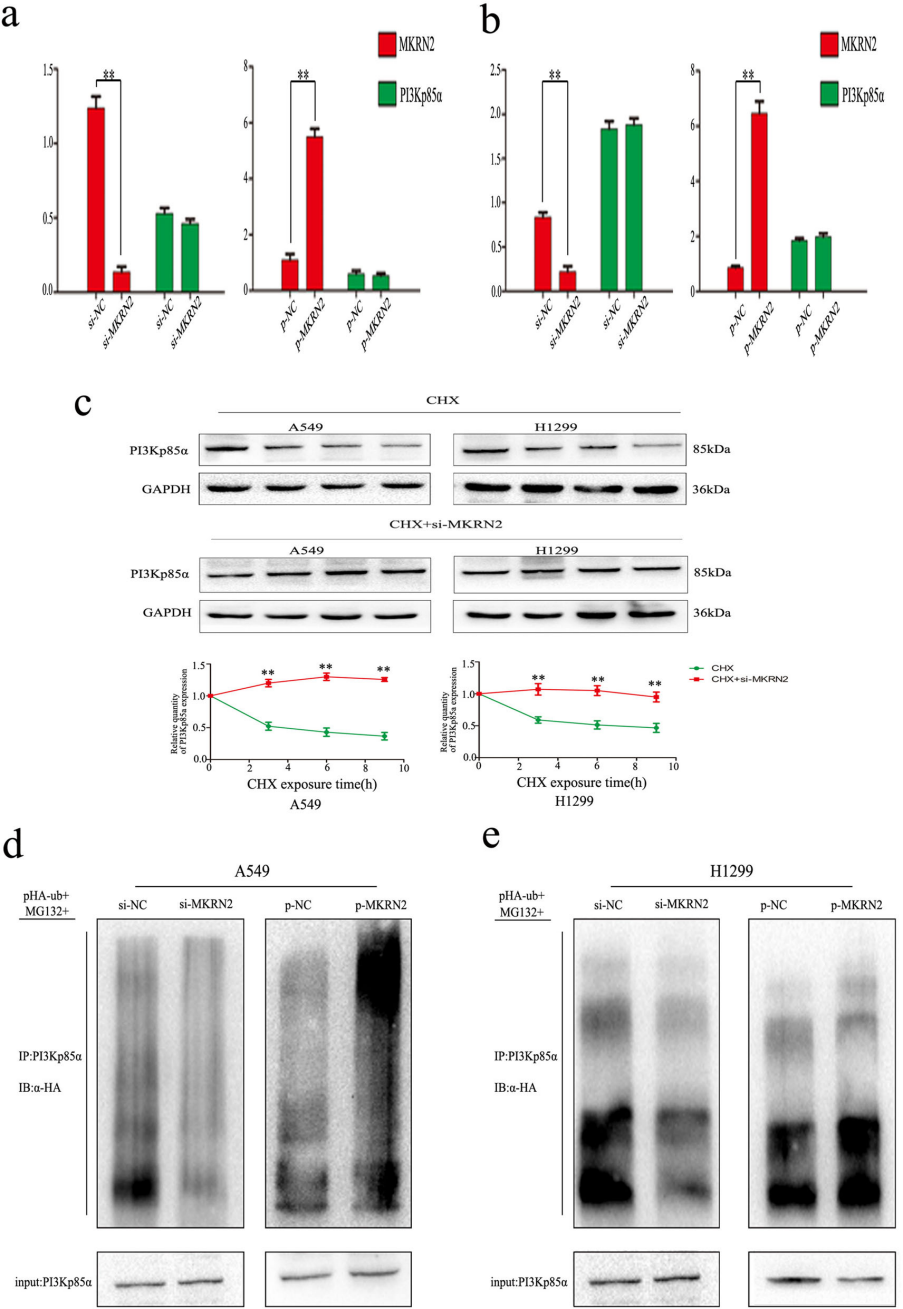
*MKRN2* mediates PI3Kp85α ubiquitination and degradation to affect the Akt pathway. **a, b** mRNA levels of the p85α subunit of PI3K in **a** A549 and **b** H1299 cells transfected with si-*MKRN2*. **c** Relative PI3Kp85α protein expression (normalized to GAPDH) as measured by densitometry. *P < 0.05; **P < 0.01. **d, e** Effects of *MKRN2* expression on PI3Kp85α ubiquitination. **d** A549 and **e** H1299 cells transfected with si-*MKRN2* or the *MKRN2*-expression plasmid along with HA-ubiquitin (Ub). Levels of PI3Kp85α ubiquitination were evaluated by immunoprecipitation using the anti-PI3Kp85α antibody, followed by anti-HA immunoblotting

We then evaluated how *MKRN2* interacts with PI3Kp85α to activate the Akt-signaling pathway to promote cancer-cell migration and invasion. Real-time PCR analysis indicated that the PI3Kp85α expression was unchanged, regardless of *MKRN2* overexpression or knockdown in both A549 and H1299 cells (Fig. 5a and b). These results indicated that the interaction between *MKRN2* and PI3Kp85α occurs at the protein level rather than the mRNA level. Accordingly, we treated A549 and H1299 cells with cycloheximide (CHX) for 0 h to 9 h as a control and with both si*-MKRN2* and CHX in the experimental group, with densitometric analysis revealing significantly higher levels of PI3Kp85α upon silencing *MKRN2* relative to controls (Fig. 5c). This result indicated that *MKRN2* expression promoted PI3Kp85α degradation. Previous studies reported that *MKRN2* exhibits E3 ubiquitin ligase activity [25, 26]. Therefore, we hypothesized that *MKRN2* might contribute to activation of the Akt pathway by attenuating PI3Kp85α ubiquitination.. Then we transfected flag-tagged *MKRN2* and si-*MKRN2* into A549 cells along with HA-ubiquitin and treated cells with the 26S proteasome inhibitor MG132. PI3Kp85α ubiquitination levels were then evaluated according to IP of PI3Kp85α using the anti-PI3Kp85α antibody, followed by anti-HA immunoblotting. The results revealed a positive correlation between *MKRN2* levels and PI3Kp85α ubiquitination (Fig. 5d) in A549 cells, with similar results obtained in H1299 cells (Fig. 5e). These data suggested that *MKRN2* was involved in the ubiquitin-dependent degradation of PI3Kp85α to activate the Akt pathway.

## Discussion

In this study, we demonstrated that *MKRN2* protein levels were correlated with a range of clinicopathological parameters, such as differentiation, lymph node metastasis, p-TNM staging, and prognosis, and primarily localized to the cytoplasm and nucleus of NSCLC tissues and cells. Furthermore, we found that *MKRN2* inhibited cell migration and invasion in NSCLC cells by regulating the expression of RhoA/ROCK1 and MMP9, thereby influencing the malignant behavior of tumor cells. Further studies are needed to evaluate the mechanisms mediating these effects.

The role of the PI3K/Akt pathway in cancer according to its effects on cell migration and invasion is well established [32–34]. In the present study, we found that *MKRN2* reduced Akt phosphorylation by attenuating PI3Kp85α levels, which confirmed that an *MKRN2* function includes regulation of PI3K/Akt signaling. Specifically, we found that *MKRN2* altered PI3Kp85α ubiquitination in NSCLC cells and promoted the ubiquitin-dependent degradation of PI3Kp85α, resulting in reduced activation of the Akt pathway and stimulation of downstream gene transcription. However, this study did not demonstrate which *MKRN2* domain is involved in the ubiquitination process or whether *MKRN2* interacted directly with PI3Kp85α to promote its ubiquitination. These details remain unclear and require further experimentation.

Our results indicated that *MKRN2* played a critical role in NSCLC by inhibiting cancer-cell metastatic potential through the PI3K/Akt-signaling pathway, suggesting *MKRN2* as a candidate prognostic biomarker and possible therapeutic target in NSCLC. Therefore, in contrast to previous studies, our findings suggested that *MKRN2* might participate in different biological mechanisms in cancer cells. Although further studies are needed to determine how *MKRN2* functions in these biological processes, our results provide important insights into the roles and mechanisms of *MKRN2* in tumorigenesis.

## Conclusions

In summary, our findings indicated that *MKRN2* played a critical role in regulating the progression of NSCLC through inhibition of cancer-cell metastatic potential. This role included *MKRN2*-specific inhibition of PI3Kp85α ubiquitination and degradation, thereby promoting activation of Akt signaling and suggesting *MKRN2* as a candidate prognostic biomarker and possible therapeutic target in NSCLC.

## Abbreviations

CHX: Cycloheximide
DMSO: Dimethyl sulfoxide
FBS: Fetal bovine serum
HA: Hemagglutinin
IP: Immunoprecipitation
MEM: Modified Eagle medium
*MKRN2*: Makorin RING zinc finger-2
MMP: Matrix metalloproteinase
NSCLC: Non-small-cell lung cancer
PBS: Phosphate-buffered saline
PI3K: Phosphatidylinositol-3 kinase
ROCK: Rho-associated protein kinase
siRNA: Small-interfering RNA
